# Galangin promotes apoptosis by upregulating the pro-apoptotic gene BAX in triple-negative breast cancer

**DOI:** 10.1186/s43046-024-00246-y

**Published:** 2024-12-20

**Authors:** Shruti Sinnarkar, Poonam Suryawanshi, Amol Dilip, Jitendra Bhawalkar, Vaibhav Ladke

**Affiliations:** 1 Dr. D. Y. Patil Biotechnology & Bioinformatics Institute, Dr. D. Y. Patil Vidyapeeth, Tathawade, Pune, India; 2 Central Research Facility, Dr. D. Y. Patil Medical College, Hospital and Research Centre, Dr. D. Y. Patil Vidyapeeth, (Deemed to Be University), Sant Tukaram Nagar, Pimpri, Pune, 411018 India; 3 Institute Of Applied Biological Research and Development, a Division of, Nirav BioSolutions Pvt Ltd , Aundh, Pune, India; 4 Dr. D. Y. Patil Medical College, Hospital and Research Centre, Dr. D. Y. Patil Vidyapeeth, (Deemed to Be University), Sant Tukaram Nagar, Pimpri, Pune, 411018 India

**Keywords:** Triple-negative breast cancer, Galangin, In silico analysis, Apoptosis, MAD MB-231

## Abstract

**Background:**

Triple-negative breast cancer (TNBC) is one of the most aggressive and formidable subtypes of breast cancer, devoid of targeted therapy and frequently leading to unfavorable prognoses and significant side effects. The demand for creative and effective treatment options has prompted the current study to investigate the potential of natural chemicals as therapeutic agents. This study intends to examine the efficacy of Galangin, a naturally occurring flavonoid, in treating triple-negative breast cancer.

**Methods:**

The research utilizes a dual methodology, combining in silico network pharmacology with in vitro experimental methods. The in silico research proved crucial in finding significant gene targets and cellular signaling pathways influenced by Galangin in triple-negative breast cancer. To corroborate these computational predictions, a variety of in vitro studies were conducted, including the MTT assay, wound scratch assay, apoptosis assay, reactive oxygen species assay, mitochondrial membrane potential assessment, and RT-PCR.

**Results:**

Fifteen prevalent genes were identified, demonstrating involvement in cellular proliferation, apoptosis regulation, cell migration, MAPK cascade regulation, and cell cycle regulation. The predominant genes implicated in the ten principal pathways were MAPK1, MAPK8, MAPK14, and IL6, which were observed to be linked to the MAPK signaling pathway, perhaps serving as the critical channel through which Galangin may facilitate the treatment of oral cancer. In vitro experiments demonstrated anti-proliferative effects, late-stage apoptosis, anti-migratory characteristics, antioxidant activity, and upregulation of the pro-apoptotic BAX gene.

**Conclusion:**

This study’s results demonstrate that Galangin possesses considerable anti-proliferative effects on TNBC cells, underscoring its potential as a viable therapeutic drug. These findings facilitate the development of more effective and precisely focused therapy approaches for TNBC, providing optimism for enhanced treatment outcomes for patients suffering from this challenging disease.

**Supplementary Information:**

The online version contains supplementary material available at 10.1186/s43046-024-00246-y.

## Introduction

Cancer is a leading cause of mortality globally and is projected to rise concomitantly with population growth and aging demographics. Every year, more than 11 lakh new cancer cases are registered in India, while the global average is more than 14 million [1]. The heightened incidence of cancer has been associated with lifestyle factors characterized by physical inactivity, tobacco use, and suboptimal dietary practices [2]. In particular, the incidence of breast cancer, along with other gynecological malignancies, is escalating, attributable to demographic shifts such as declining birth rates and delayed childbearing [3]. Various factors contribute to the risk of developing breast cancer, including weight gain post-adolescence, obesity, menopausal hormone therapy (MHT), lack of physical activity, alcohol consumption, and specific reproductive and hormonal conditions. These conditions include a prolonged menstrual history, recent use of oral contraceptives, and null parity. Conversely, breastfeeding has been shown to reduce the risk of breast cancer [4].

Gynecological cancers account for about 10% of cancer diagnoses in women and are a significant cause of mortality. However, breast cancer is the most prevalent malignancy among women, constituting one in four female cancer cases globally. In 2020, there were 2.3 million new breast cancer cases, with 685,000 deaths, representing 16% of female cancer fatalities [5, 6].

As previously stated, triple-negative breast cancer (TNBC) is a particularly aggressive form of breast cancer defined by the lack of expression of all three receptors: estrogen receptor (ER), progesterone receptor (PR), and human epidermal growth factor receptor-2 (HER2/ERBB2). TNBC, constituting 10–15% of breast cancer cases, is characterized by high metastatic potential and heterogeneity, leading to a notably poor prognosis and increased likelihood of relapse within 5 years following treatment, in contrast to non-TNBC cases [7]. Treating triple-negative breast cancer (TNBC) presents challenges due to its aggressive nature and lack of targeted therapies. Chemotherapy, particularly with anthracycline/taxanes, is standard but often causes significant side effects [8]. Current chemotherapeutic alternatives for breast cancer comprise agents such as tamoxifen and the cytotoxic medicines paclitaxel and docetaxel, both of which exhibit significant adverse effects [9]. Consequently, there is an emphasis on novel chemotherapeutic and chemopreventive drugs that exhibit minimal toxicity to normal tissue and offer a more advantageous treatment window.

Recent studies indicate favorable outcomes in employing dietary flavonoids for the prevention and treatment of several cancers, including gynecological malignancies. Therefore, additional investigation into the influence of dietary flavonols on cancer progression is essential to augment our comprehension and boost treatment results in gynecological cancers [10]. Flavonoids, including kaempferol, myricetin, quercetin, fisetin, Galangin, isorhamnetin, and morin, exhibit potential as therapeutic and chemopreventive medicines for gynecological and breast malignancies, with preclinical trials demonstrating favorable results [2, 11]. Through the use of in silico analysis, a number of novel flavonoid compounds have been hypothesized to possess anticancer properties. These compounds may be taken into consideration for future in vitro validation and clinical use [12–14].

Galangin (3,5,7-trihydroxyflavone), a flavonoid present in linden, propolis, Alpinia officinarum, and various other medicinal plants, demonstrates considerable anticancer efficacy via chemopreventive and therapeutic pathways against cancers including renal, hepatic, and gastric malignancies. It has exhibited pharmacological actions, encompassing apoptotic, anti-inflammatory, anticancer [15], organ-protective, and antidiabetic activities [2, 16].

Network pharmacology, a novel paradigm in drug discovery, presents a viable approach to tackling the intricacies of cancer. Conventional approaches have faced challenges in addressing the interrelated networks that underpin cancer progression. Utilizing computational biology and network analysis, network pharmacology facilitates the strategic targeting of dysregulated signaling pathways in cancer. By modeling drug-target interactions inside cellular networks, it seeks to identify optimal therapeutic targets and combinations to inhibit cancer proliferation. This strategy has the potential to address drug resistance and reduce adverse effects by targeting the disease network at a systems level through synergistic interactions. Computational models and algorithms are essential in identifying prospective drug combinations, hence aiding the development of successful multi-target cancer therapies [17, 18]. There are various limitations of computational research that need to be taken into consideration whenever any in silico studies are being carried out [19].

**Fig. 1:**
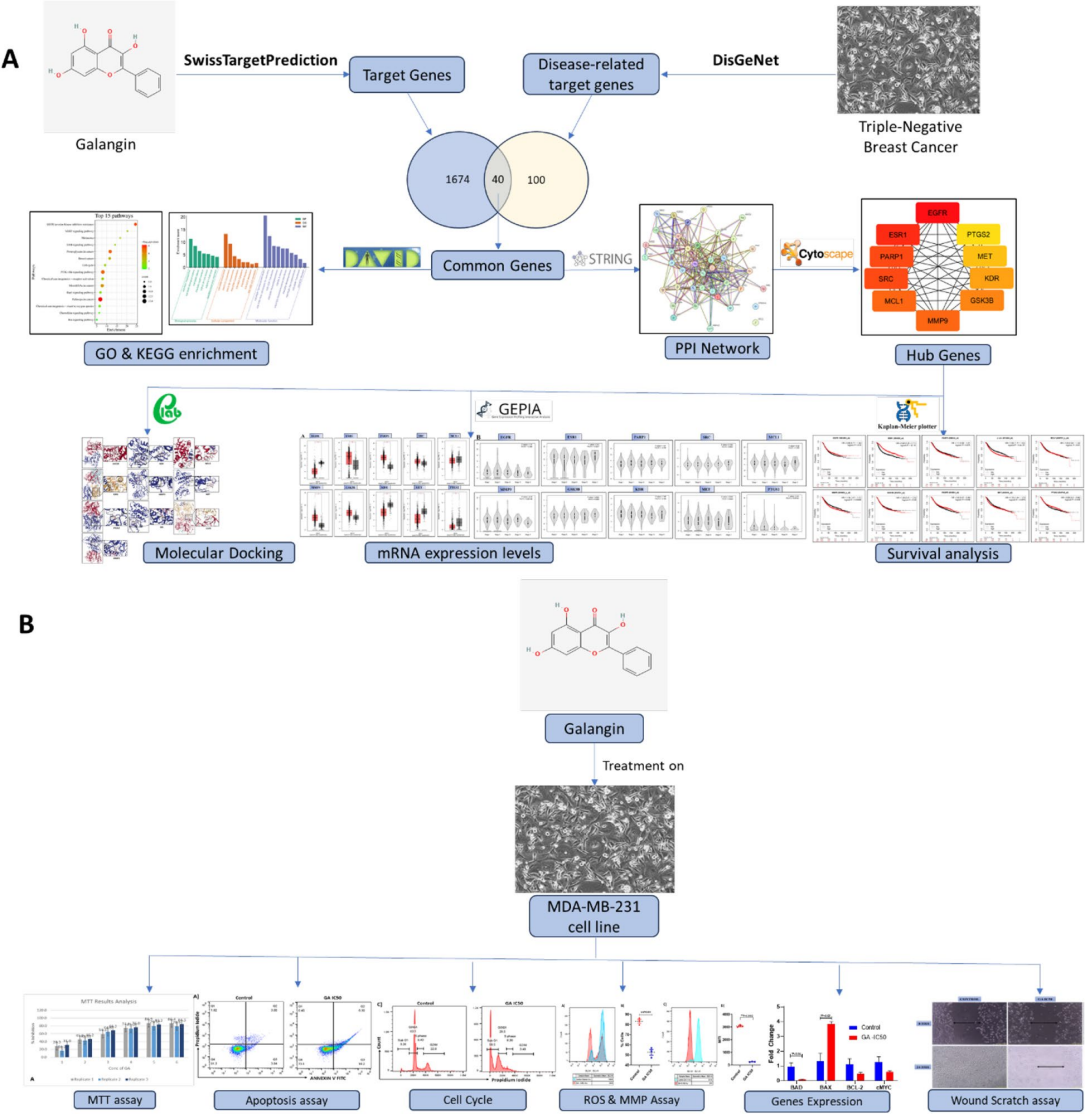
The flowchart of this study is based on (**A**) in silico network pharmacology and (**B**) in vitro assays for deciphering the potential mechanisms of Galangin against triple-negative breast cancer cell line

This study seeks to employ in silico network pharmacology to identify the primary gene targets and cellular signaling pathways influenced by Galangin in triple-negative breast cancer (TNBC). Network pharmacology is a viable approach to tackle the intricacies of cancer by addressing the interrelated networks that underpin cancer progression, hence potentially mitigating medication resistance and reducing adverse effects. Additionally, we augmented this computational strategy with in vitro techniques, including several experiments to examine Galangin’s cytotoxic, anti-migratory, and apoptotic effects on TNBC cells. The comprehensive progression of the work is illustrated in Fig. 1. This work intends to validate the anticancer properties of Galangin and elucidate its mode of action via hypothesized pathways and gene targets, thereby facilitating future therapeutic advancements.

## Methodology

This study was conducted in two phases. Initially, an in silico analysis was carried out, followed by an in vitro investigation. Approval for this study was granted by the Institutional Scientific Committee.

### In-silico analysis

#### Prediction of Drug‑likeness of Galangin

In the context of oral pharmaceuticals for humans, Lipinski's Rule of Five (RO5) was used to assess how similar Galangin was to other drugs. A large number of parameters were examined. The SwissADME server (http://www.swissadme.ch) is an online resource that calculates parameters such as oral bioavailability (OB), drug-likeness (DL), and absorption, distribution, metabolism, and excretion (ADME). The SMILES style formula for Galangin C1=CC=C(C=C1)C2=C(C(=O)C3=C(C=C(C=C3O2)O)O)O was used in SwissADME for further analysis [20].

#### Identification of potential targets for Galangin and Breast cancer

The “Swiss Target Prediction database (http://www.swisstargetprediction.ch/)” [21] was utilized to forecast the genes related to Galangin. The DisGeNet database was employed to acquire the genes linked to triple-negative breast cancer, which were subsequently contrasted with the genes connected with Galangin. The shared genes were later discovered and chosen for additional investigation.

#### Functional similarity and pathway enrichment evaluation

The Gene Ontology (GO) and Kyoto Encyclopaedia of Genes and Genomes (KEGG) pathway enrichment analyses were conducted using the Database for Annotation, Visualization, and Integrated Discovery (DAVID, https://david.ncifcrf.gov/, ver. 6.8) and the ShinyGO database, respectively, (http://bioinformatics.sdstate.edu/). Annotating and interpreting gene lists is made easier with DAVID, while Gene Ontology and pathway enrichment analysis are the focus of the ShinyGO database. On the other hand, KEGG maps metabolic pathways graphically [22, 23] and is a comprehensive route database. An extensive resource for functional genomics, the Gene Ontology (GO) provides descriptions and classifications of gene functions, among other things [24]. Careful consideration of clinical and pathological data allowed for the selection of relevant pathways linked to breast cancer. Data visualization and analysis were carried out utilizing the Bioinformatics cloud platform, an online resource for processing and presenting data (http://www.bioinformatics.com.cn/).

#### Constructing a protein-protein interaction (PPI) network

Understanding the intricate systems at work in a live cell requires an appreciation of protein-protein interaction (PPI) [25]. PPI is fundamental to all biological processes. In order to outline the protein-protein interaction (PPI) network, the goal of this analysis was to examine the cluster of target genes using the STRING database (http://string-db.org/; version 11.5). The study only included "Homo sapiens," and a confidence level of >0.9 was employed to ensure accurate results. A popular bioinformatics program that allows data visualization and integration, Cytoscape (https://cytoscape.org/; version 3.10.1) was used to generate the PPI network [26]. To find clusters or highly linked areas in the PPI network, we used the Cytoscape plugin cytoHubba (https://apps.cytoscape.org/apps/cytohubba; version 0.1). The network identified the proteins with the highest Degree values as potential targets.

#### Molecular docking analysis of Galangin

Molecular docking was conducted to investigate the interaction between the candidate proteins (hub genes) and Galangin in further detail. The purpose of the docking was to assess the interactions between the hub targets and Galangin. Molecular docking was performed with CB-Dock (http://cao.labshare.cn/cb-dock), a tool proficient in autonomously finding active sites within a specified protein, determining their centers and dimensions, and modifying the grid box size according to the query ligands [27]. The Protein Data Bank (http://www.rcsb.org) was utilized to obtain the target protein crystal. Likewise, the 3D structure of Galangin was acquired from the PubChem compound database (https://pubchem.ncbi.nlm.nih.gov/). The protein and ligand structures served as inputs for CB-Dock, where docking analysis was conducted to examine the binding interactions between the proteins and Galangin.

### Analysis of gene expression levels and overall survival of hub genes

This work utilized Gene Expression Profiling Interactive Analysis (GEPIA; http://gepia2.cancer-pku.cn/) to confirm the differential expression of hub genes in breast cancer and normal breast tissues. GEPIA is an online platform that provides interactive and customisable features using data from The Cancer Genome Atlas (TCGA) and the Genotype-Tissue Expression (GTEx) database. Moreover, GEPIA enabled the examination of these genes according to pathological stages, yielding significant insights into their expression patterns across various illness stages [28]. The Kaplan-Meier Plotter (http://kmplot.com/analysis/index.php?p=service) [29], a cancer genomics dataset, was employed to examine the influence of hub targets on the overall survival (OS) of breast cancer patients. This dataset facilitates the evaluation of the prognostic relevance of genes on survival outcomes. Patients with breast cancer were classified into two groups according to the expression levels of the hub genes: low and high expression. A Kaplan-Meier survival plot was created to compare the survival outcomes of the two groups. Moreover, hazard ratios (HR) together with their respective 95% confidence intervals and log-rank *P*-values were computed for additional statistical significance evaluation.

### Galangin's anticancer properties

#### Cell culture and maintenance

The MDAMB231 cell line, associated with triple-negative breast cancer, was acquired from the National Centre for Cell Sciences (NCCS) in Pune. The cells were grown in MEM (Gibco) medium at 37°C. The culture medium was augmented with 10% fetal bovine serum (FBS, Gibco™) and 1% antimycotic-antibiotic solution (Gibco™). The cells were sustained in a CO2-enriched atmosphere with a concentration of 5% to promote their growth and viability.

#### Galangin stock solution preparation

Galangin was prepared for cytotoxicity testing on the MDAMB231 cell line as follows. A stock solution with a concentration of 10 mg/mL was prepared by dissolving 10 mg of Galangin in 1 mL of DMSO. The stock solution was subsequently held at ambient temperature. A working solution with a concentration of 1 mg/ml was generated from the stock solution by diluting it in complete medium and filtering it through a sterile 0.22 µm filter to assure sterility. Dilutions of the chemical were prepared in full culture medium to achieve concentrations of 80, 60, 40, 20, 10, and 5 µg/mL. The final concentration of DMSO utilized in the experiments was maintained below 0.1%. Carboplatin, a reference chemotherapeutic agent with proven effectiveness in cancer therapy, was utilized.

### MTT assay

Cell cytotoxicity was assessed using the MTT conversion assay. The density of MDAMB231 cells in a 96-well plate was 5 × 10^4^ cells/mL. Different concentrations of Galangin, ranging from 5 µg/mL to 80 µg/mL, were used to treat the cells. Besides Galangin, the standard drug carboplatin was given at doses ranging from 10 to 80 µg/mL. Each well was incubated for 4 hours after 10 µL of a 5 mg/mL MTT solution was added after the treatment. To dissolve the formazan crystals, 100 µL of dimethyl sulfoxide (DMSO) was added to every well. Using the Multiskan SkyHigh plate reader from Thermo Fisher Scientific, the absorbance at 570 nm was measured. This wavelength represents the amount of purple formazan that was formed and indicates the presence of living cells. The IC50 values were calculated using an Excel spreadsheet in Microsoft Office, and this value was used in the following research.

### Apoptosis analysis

The apoptosis experiment was conducted utilizing the FITC Annexin V/Dead Cell Apoptosis Kit (Invitrogen-Molecular Probes®). Following 24 h of treatment, the MDAMB231 cells were rinsed with cold 1 × PBS. The untreated cells served as negative controls for the apoptosis assay. Flow cytometry was employed to analyze these labeled cells at emission wavelengths of 530 nm and greater than 575 nm [30].

### Cell cycle analysis

MDA-MB231 cells were collected 24 hours after being washed with 1X PBS and trypsinized, regardless of whether they had been treated or not with Galangin and carboplatin. The collected cells were treated with a mixture that included 25 μL of RNase A (20 mg/ml, Invitrogen), 2 mM MgCl2 (Sigma), and 5-10 μL of propidium iodide (100 μg/ml, Invitrogen). The cells were incubated at room temperature for a duration of 10-15 minutes before being analyzed using a BD Bioscience FACS-calibre instrument.

### Reactive oxygen species (ROS)

ROS generation was assessed by DCFHDA and flow cytometry [31]. In this assay, MDA-MB231 cells were cultured for 24 hours in 6-well plates. The cells were treated with galangin and carboplatin at doses equal to the IC50 value after reaching 70–80% confluence. Using the Beckman Coulter Cytomics FC 500 equipment and flow cytometry, fluorescence was measured at wavelengths of 495 nm and 520 nm.

### Analysis of Mitochondrial membrane potential (ΔΨm)

We assessed ΔΨm in MDA-MB231 cells using the MitoProbe™ DiIC1 (5) Assay Kit in accordance with the manufacturer's instructions. This package contains CCCP, a mitochondrial membrane potential disruptor used in research, and DiIC1 (5), a cyanine dye sensitive to changes in membrane potential. Strong far-red fluorescence can be produced by DiIC1 (5) easily penetrating cell cytoplasm and concentrating in mitochondria with active ΔΨm.

### Anti-migratory activity by Wound scratch assay

To investigate cell invasion, MDAMB231 cells cultured with Galangin underwent an anti-migration test. 24-well plates were injected with a density of 2 × 10^5^ cells/ml and allowed to reach confluency of greater than 90%. A 200 μL plastic pipette tip was used to make a linear incision in the middle of each well. Before a 24-hour incubation period, the scratched cell monolayers were treated with Galangin and a control group after three washing with 1X PBS to remove cell debris. A Magnus INVI phase contrast microscope was used to take pictures of the scratches at 0 and 24 hours. The cells were then allowed to migrate by being incubated for 24 hours at 37°C in medium containing 5% serum, either with or without the medications [32].

### RNA isolation and RT-PCR

The doses of the drug that were calculated were given to the cells in culture. Following drug incubation, cells were harvested by removing the growth media. Total RNA was extracted from the cells following the manufacturer's instructions using the TRIzol method (TRIzol™ Reagent, Invitrogen cat no. 15596018). The primers that were used in this study are listed in Supplementary Table 1 [ST: 1].

### Statistical analysis

The data are presented as the Mean ± Standard Deviation (SD), and the studies were carried out three times. GraphPad Prism 8 was used to analyze the data. The two-way ANOVA was used to determine the statistical significance of the groups in comparison to the control, with the relevant post hoc tests performed at ****P < 0.0001, ***P < 0.001, and **P < 0.01.

## Results

### In silico analysis

#### Galangin exhibited drug likeliness property

It follows from our findings that Galangin follows Lipinski's Rule of Five (RO5). Adhering to Lipinski's Rule of Five, a molecule with medicinal potential must have the following characteristics: a polar surface area (PSA) of 140 Å² or smaller, a molecular weight (MW) below 500 g/mol, and a computed octanol/water partition coefficient (XLogP3) below 5. For the best drug-ligand interactions, these three qualities are crucial. In addition, Galangin has a maximum of 10 hydrogen bond acceptors and no more than 5 hydrogen bond donors, with less than 10 rotatable bonds. Galangin possesses beneficial drug-like properties since its chemical characteristics meet the RO5 requirements.

#### Target identification and analysis

**Fig. 2:**
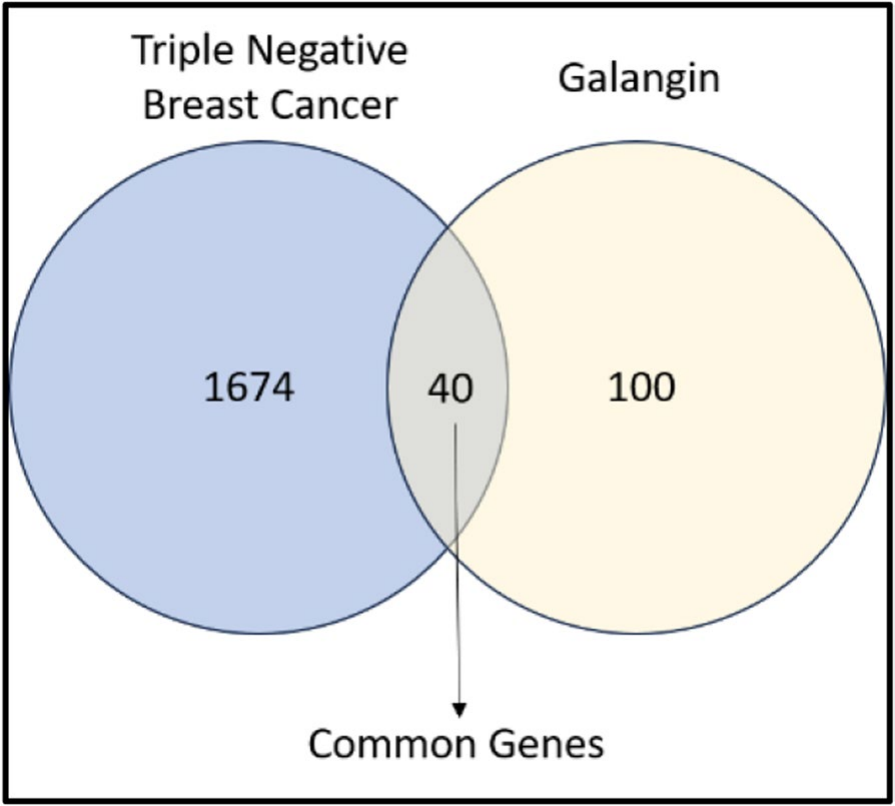
Number of genes common between triple-negative breast cancer and Galangin

This study used the DisGeNet database to find targets related to triple-negative breast cancer. The search keyword used was "Triple-negative breast cancer." There were 1,674 targets associated with triple-negative breast cancer that were found during the screening. One hundred Galangin targets were found by searching the Swiss Target Prediction database. Forty genes were found to be shared between the Galangin targets and the triple-negative breast cancer targets (Fig. 2).

### The establishment of a PPI network and the identification of top hub genes

**Fig. 3:**
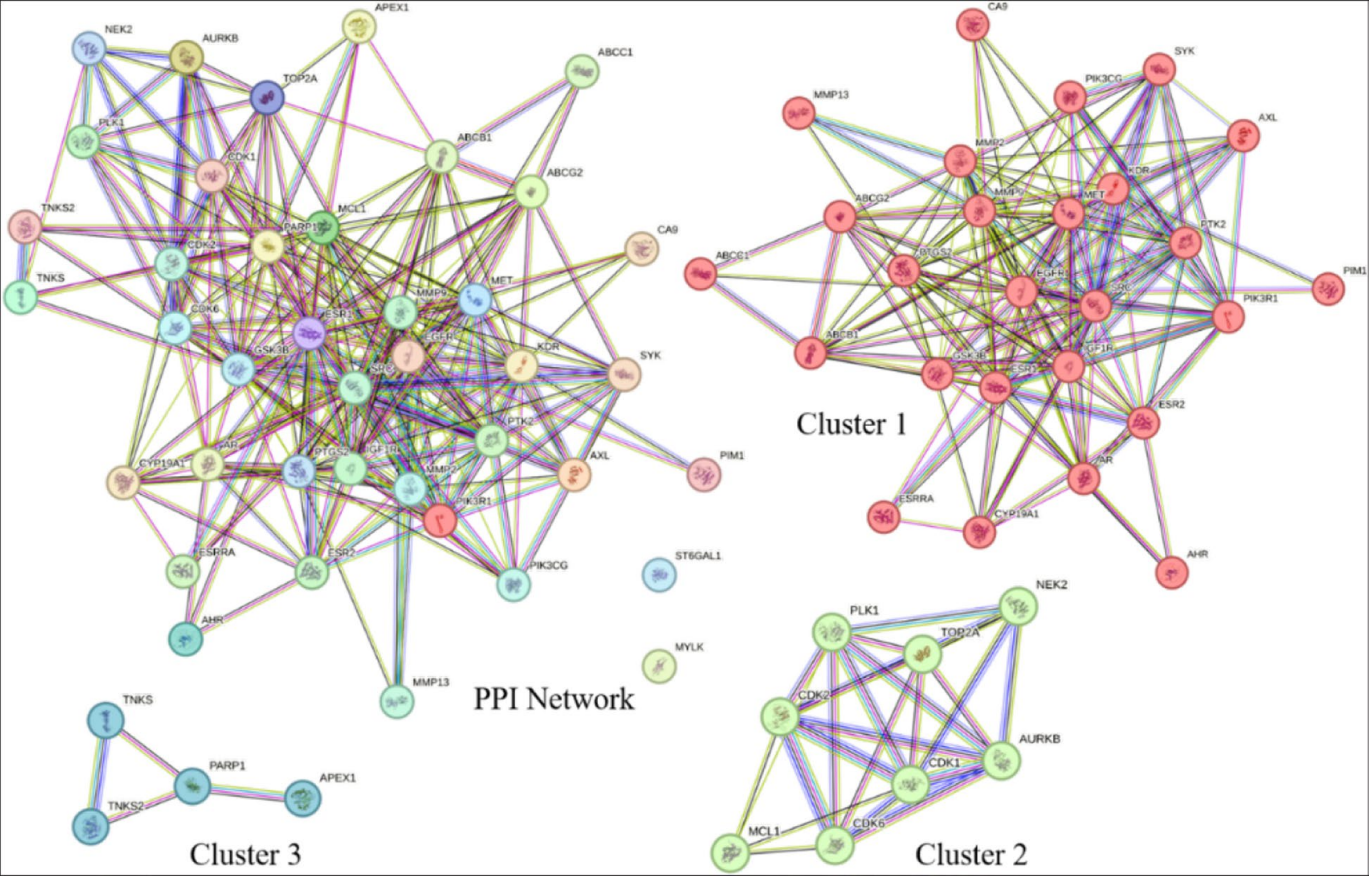
Protein–protein interaction network comprising 40 genes: cluster 1 has 26 proteins (nodes) interconnected by 155 edges, with a PPI enrichment *p*-value of 1.0e − 16. Cluster 2 has 8 proteins (nodes) interconnected by 24 edges, with a PPI enrichment *p*-value of 6.46e − 11. Cluster 3 has 4 proteins (nodes) and their interconnections (four edges), (PPI enrichment *p*-value: 0.000233)

To examine the interplay between the designated targets' proteins, the study made use of the STRING Database. Clusterone in Cytoscape 3.10.1 was used to conduct further analysis on the PPI network in order to identify modules. The network, which includes 40 shared genes, showed a *p*-value of less than 1.0e-16, indicating that the proteins in this network interact more strongly with each other than a randomly selected set of proteins from the genome with similar size and degree distribution. The proteins are shown to be biologically connected as a cohesive unit in Fig. 3, which is supported by this enrichment. Three distinct clusters were identified in the investigation. The first cluster exhibited a *p*-value of less than 0.05 (*p*-value – 1.0e-16), the second cluster showed a *p*-value of 6.46e-11, and the third cluster noted a *p*-value of 0.000233. The *p*-values indicate that the target proteins within each cluster are significantly connected and likely share similar biological functions. Cluster 1 (Fig. 3) was associated with the positive control of cellular proliferation, apoptosis, cell migration, and the MAPK signaling cascade. In contrast, Cluster 2 (Fig. 3) was linked to cell division, the positive control of the G2/M transition in the mitotic cell cycle, and the negative regulation of the G1/S transition in the mitotic cell cycle.

**Fig. 4:**
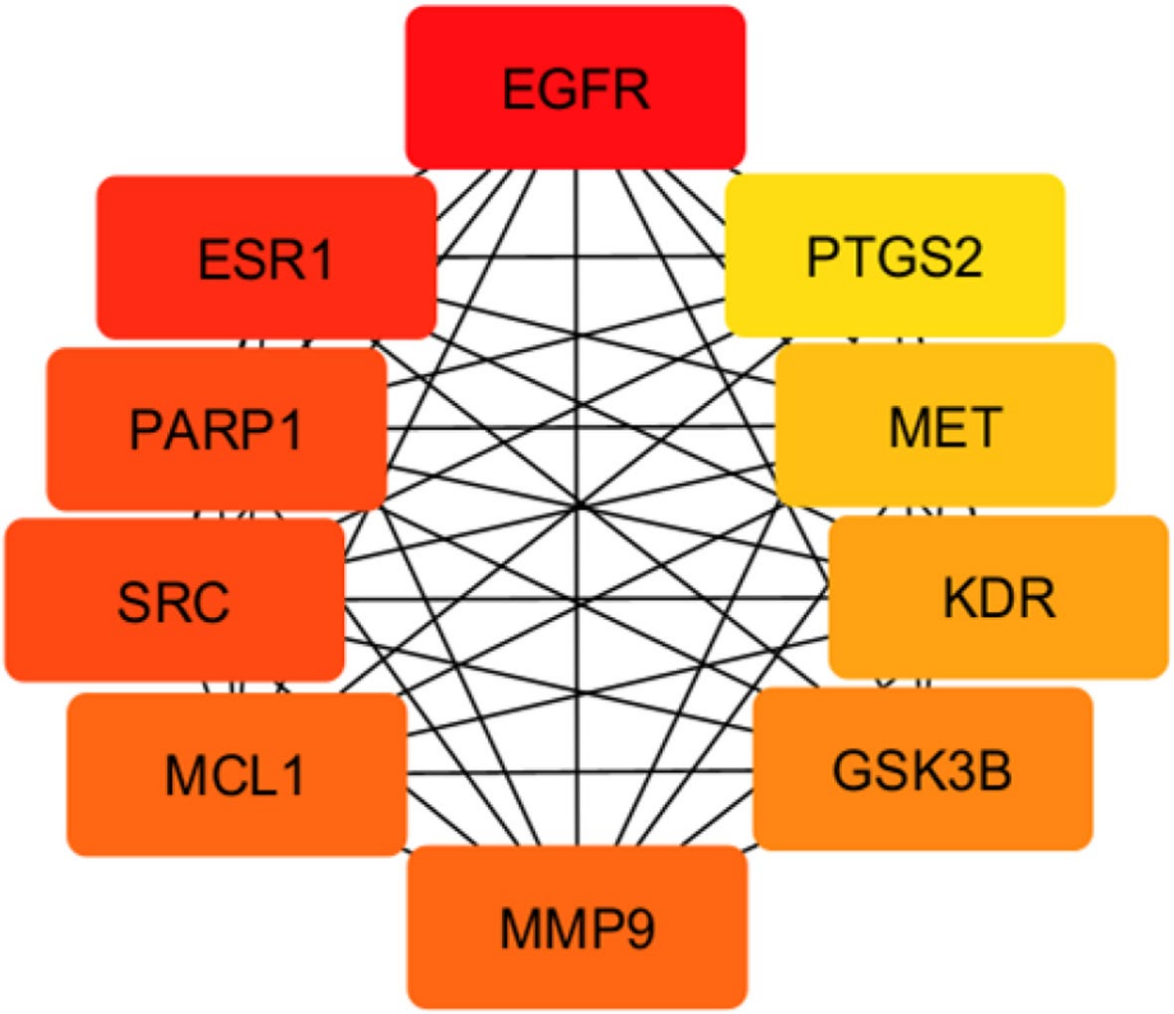
Figure showing the top 10 hub genes from common 40 genes

Using a variety of techniques, the study determined that EGFR, ESR1, PARP1, SRC, MCL1, MMP9, GSK3B, KDR, MET, and PTGS2 were the ten main hub genes. The most highly active gene among these hub genes was found to be EGFR. The study identified ten key hub genes: EGFR, ESR1, PARP1, SRC, MCL1, MMP9, GSK3B, KDR, MET, and PTGS2. It used a variety of methods to reach this conclusion. The EGFR gene was determined to be the most active of these hub genes (Table 1, Fig. 4).

**Table 1:** Various algorithms used to determine the top 10 hub genes based on CytoHubba

Algorithms	MCC	MNC	Degree
GENES	EGFR	EGFR	EGFR
	ESR1	ESR1	ESR1
	MMP9	PARP1	PARP1
	SRC	SRC	SRC
	IGF1R	MCL1	MCL1
	PARP1	MMP9	MMP9
	MET	GSK3B	GSK3B
	MCL1	KDR	KDR
	MMP2	MET	MET
	ABCB1	PTGS2	PTGS2

### Functional enrichment analysis

**Fig. 5:**
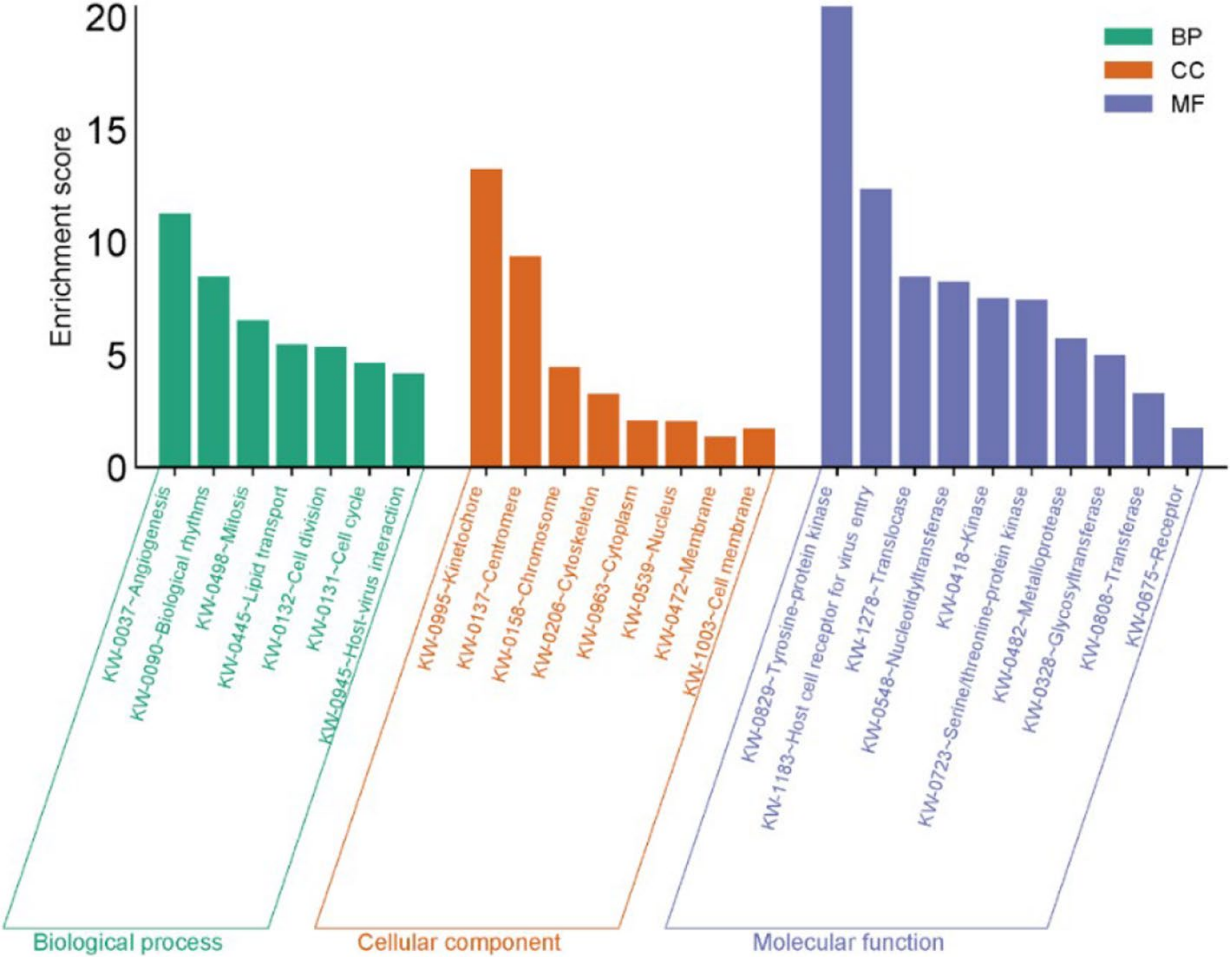
GO enrichment analysis was conducted by selecting 7 biological processes (BP), 8 molecular functions (MF), and 10 cellular components (CC) from the DAVID Database for the targets associated with Galangin in the treatment of breast cancer

Approximately twenty-five GO terms were discovered in an analysis of the forty genes that were found to be common. Angiogenesis, biological rhythms, mitosis, and lipid transport are all biological processes (BP) that these targets are involved in, according to the research. The kinetochore, centromere, chromosome, and cytoskeleton were all part of the cellular component (CC) findings. When it comes to molecular function (MF), the targets primarily participate in activities such as tyrosine-protein kinase, translocase, viral entry mediated by host cell receptors, and serine/threonine-protein kinase (Fig. 5).

### KEGG enrichment analysis

**Fig. 6:**
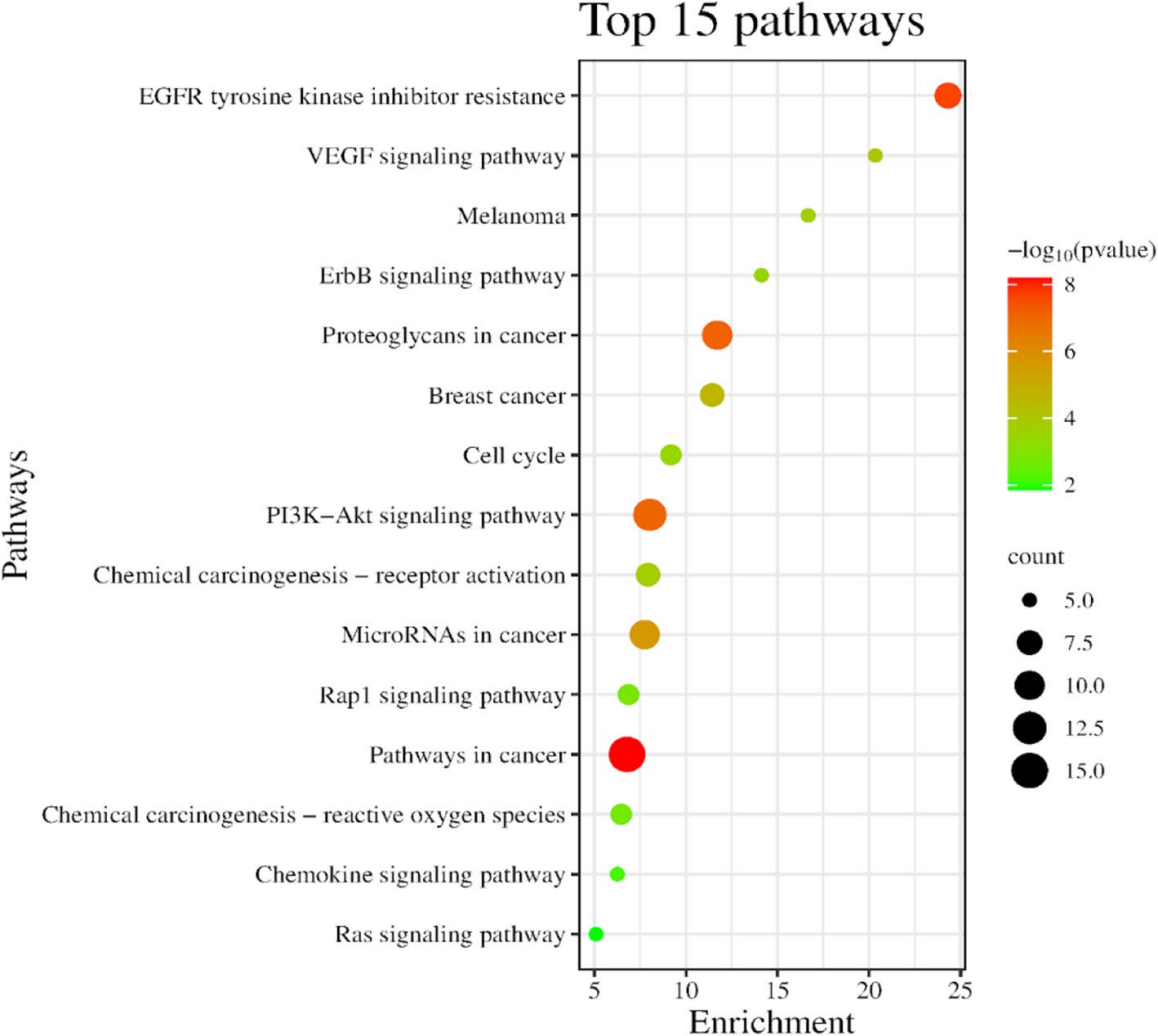
A Bubble plot illustrating the enrichment of the top 15 signaling pathways related to oral cancer was created. The *X*-axis denotes the enrichment factor of the genes, and the *Y*-axis signifies the various pathways. The circles in the graphic are color-coded and sized according to the Log10 (*p*-value), with red representing pathways containing the highest gene counts and light green denoting pathways with fewer genes

The KEGG pathway analysis yielded 75 pathways in total. Further analysis was limited to the top fifteen pathways. Pathways associated to cancer, microRNAs in cancer, proteoglycans in cancer, resistance to EGFR tyrosine kinase inhibitors, and the PI3K-Akt signaling pathway were among the many that the ten main hub genes showed substantial associations with, according to the analysis (Fig. 6).

The genes EGFR, PIK3R1, MET, GSK3B, SRC, and CDK6 were consistently found to be involved in the 10 major pathways. These genes showed significant associations with the PI3K-Akt signaling system, which is important in regulating a variety of functions in cells that influence carcinogenesis, such as metabolic pathways, cell survival, cell proliferation, gene expression, and protein synthesis [33]. Table 2 shows that Galangin may play a role in breast cancer treatment, with the PI3K-Akt signaling pathway as a key focus and apoptosis as the predominant cellular mechanism.

**Table 2:** Top 10 pathways showing the genes involved

Pathway term ID	Fold enrichment	*p*-value	Gene count	Genes
hsa05200:Pathways in cancer	6.782799749	6.43E − 09	15	GSK3B, MMP2, PIK3R1, PTGS2, ESR1, MMP9, EGFR, PTK2, ESR2, IGF1R, AR, CDK6, CDK2, PIM1, MET
hsa04151:PI3K-Akt signaling pathway	8.025998143	9.26E − 08	12	GSK3B, CDK6, SYK, CDK2, KDR, PIK3R1, MET, PTK2, EGFR, PIK3CG, IGF1R, MCL1
hsa05206:MicroRNAs in cancer	7.745519713	2.71E − 06	10	ABCC1, ABCB1, CDK6, PIM1, PIK3R1, PTGS2, MET, MMP9, EGFR, MCL1
hsa05205:Proteoglycans in cancer	11.71273713	8.24E − 08	10	SRC, MMP2, KDR, PIK3R1, ESR1, MET, MMP9, PTK2, EGFR, IGF1R
hsa01521:EGFR tyrosine kinase inhibitor resistance	24.31504923	2.23E − 08	8	GSK3B, SRC, AXL, KDR, PIK3R1, MET, EGFR, IGF1R
hsa05207:Chemical carcinogenesis—receptor activation	7.928197065	1.82E − 04	7	AR, SRC, AHR, PIK3R1, ESR1, EGFR, ESR2
hsa05224:Breast cancer	11.43386243	2.36E − 05	7	GSK3B, CDK6, PIK3R1, ESR1, EGFR, ESR2, IGF1R
hsa04110:Cell cycle	9.176220807	3.88E − 04	6	GSK3B, CDK6, PLK1, CDK2, CDK1, AURKB
hsa04015:Rap1 signaling pathway	6.86031746	0.001446	6	SRC, KDR, PIK3R1, MET, EGFR, IGF1R
hsa05208:Chemical carcinogenesis-reactive oxygen species	6.46038864	0.001886	6	SRC, AHR, PIK3R1, MET, PTK2, EGFR

Phosphoinositide 3-kinase-Akt signaling pathway PIK3CA is a constituent of the PI3K signaling pathway, often dysregulated in numerous cancer types, and is implicated in cell growth, proliferation, differentiation, protein synthesis, and apoptosis [34]. Somatic mutations in PIK3CA are deemed responsible for the enhancement of its kinase activity, leading to cellular transformation [35]. Phosphoinositide 3-kinases (PI3Ks) are enzymes crucial for cellular development and differentiation. This is initiated by receptor tyrosine kinases (RTKs) such as epithelial growth factor receptor (EGFR). Protein kinase B (PKB or AKT) is phosphorylated and activated subsequent to the activation of PI3K, followed by its translocation to the cell membrane [36]. AKT can activate the mammalian Target of Rapamycin (mTOR) complexes (mTORC1 and mTORC2), leading to the transcription of PI3K pathway signaling molecules, such as serine/threonine protein kinase SGK146. This consequently results in enhanced protein synthesis and cellular proliferation [37]. The PI3K/AKT pathway is enhanced by IGF-1, EGF, and sonic hedgehog (SHH) [38], and is suppressed by several factors, including phosphatase and tensin homolog (PTEN). Alterations and mutations in the PTEN/PI3K/AKT/mTOR pathway enhance cellular proliferation and diminish apoptosis [39]. These mutations have been associated with drug resistance in the management of oral cancer. Drug resistance is believed to result from modified activation of the MDR-1 gene and extended cellular viability. The predominant mutational sites in oral cancer are located in exons 9 and 20 of the PIK3CA gene. E542K, E545K, H1047R, H1047Y, and H1048Q are prevalent variants in this gene [40].

### Hub target confirmation using molecular docking

**Fig. 7:**
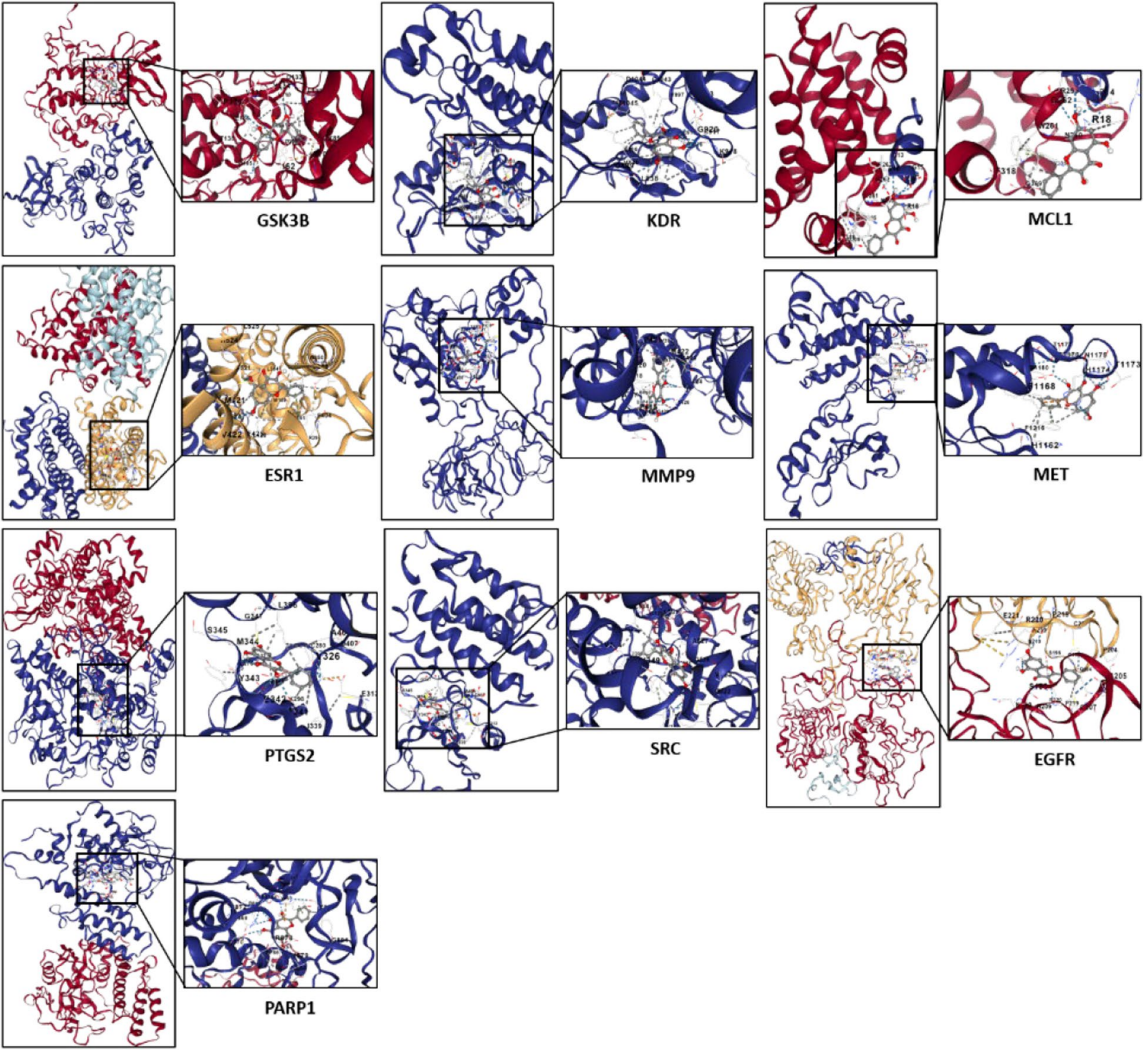
Sketch and matching diagrams of molecular docking of Galangin and top 10 hub genes (target proteins)

To assess the reliability of the drug-target interactions, ten hub genes were selected as targets for molecular docking studies. The structure of Galangin was submitted to CB-DOCK to evaluate its docking affinity with EGFR, ESR1, PARP1, SRC, MCL1, MMP9, GSK3B, KDR, MET, and PTGS2. A reduced energy value signifies a more stable conformation of the ligand binding to the receptor, implying an increased probability of contact. This investigation revealed that the binding energies between Galangin and the core target proteins were below − 5.0, signifying a robust binding affinity between Galangin and the core targets. The binding energies are detailed in Table 3, while the docking schematic representations of the interactions between the target proteins and Galangin are shown in Fig. 7.

**Table 3:** Molecular docking scores of Galangin and hub target proteins

Receptor	PDB ID	Binding energy kcal/Mol
EGFR	1IVO	− 7.8
ESR1	3OSA	− 8.3
PARP1	1UK1	− 9.2
SRC	6ATE	− 9.5
MCL1	3MK8	− 6.3
MMP9	1L6J	− 9.1
GSK3B	1Q4L	− 9.3
KDR	2OH4	− 9.4
MET	1R1W	− 7.2
PTGS2	5F19	− 9.8

### External validation of hub genes

#### mRNA expression levels of hub genes

**Fig. 8:**
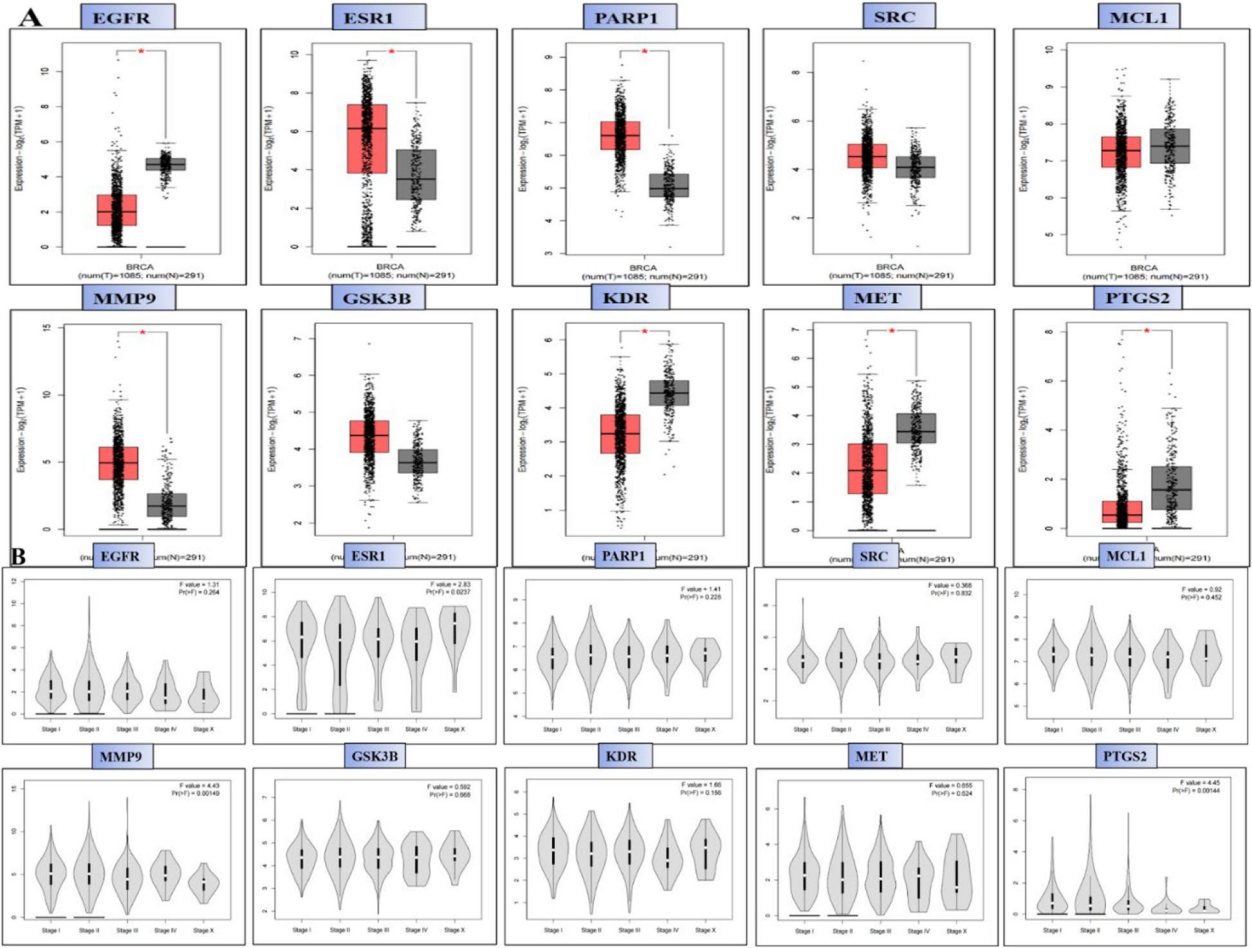
The mRNA expression levels of hub genes in The Cancer Genome Atlas (TCGA) and Genotype-Tissue Expression (GTEx) datasets. **A** mRNA expression levels in the GEPIA database (Boxplot of hub genes). Red represents breast cancer tissue, whereas grey signifies normal breast tissue. **B** mRNA expression levels and disease stages in the GEPIA database. (Stage plot of hub genes)

We employed the GEPIA database to analyze the differential expression of hub genes in breast cancer and normal tissues. Our study demonstrated that mRNA levels of ESR1, PARP1, GSK3B, and MMP9 were considerably increased in breast cancer tissues relative to normal samples (*p* < 0.01) (Fig. 8A). Additionally, we examined the correlation between mRNA levels of the hub genes and the pathological stages of breast cancer. The findings demonstrated that the expression levels of EGFR, MMP9, ESR1, MET, and PTGS2 showed considerable variations throughout various clinical phases. Notably, EGFR, MMP9, ESR1, and PTGS2 exhibited significant elevations in stage II (Fig. 8B). The results indicate that the expression levels of these five genes may correlate with the advancement of breast cancer.

#### Survival analysis of the hub genes

**Fig. 9:**
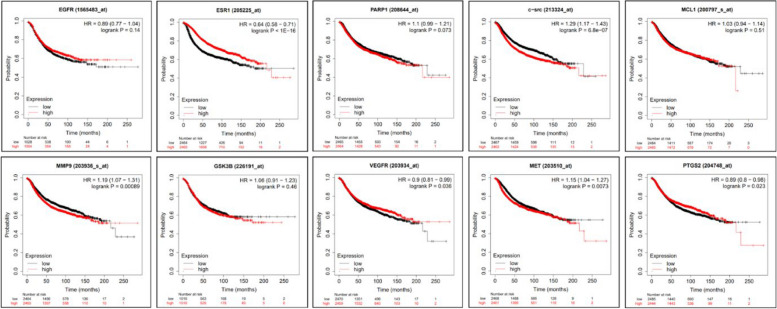
Kaplan–Meier overall survival analyses of patients with breast cancer based on the expression of the ten hub genes. HR, hazard ratio ("http://kmplot.com/analysis/index.php?p=service&cancer=pancancer_rnaseq")

A survival analysis was carried out on the ten hub genes, which are as follows: EGFR, ESR1, PARP1, SRC, MCL1, MMP9, GSK3B, KDR, MET, and PTGS2. Four thousand nine hundred and twenty-nine breast cancer patients from the TCGA database were used in the investigation. The results of the study demonstrated that every hub gene exhibited a significant association with a poor prognosis or outcome (*p* < 0.05, Fig. 9).

### Anticancer activity of Galangin on MDA-MB231 cell line

#### Cytotoxic effect of Galangin on MDA-MB231 cells

Various dosages of Galangin (5, 10, 20, 40, 60, and 80 µg/ml) and Carboplatin (10, 20, 40, and 80 µg/ml) exhibited a significant cytotoxic effect on the MDAMB231 cell line in a dose-dependent manner, with an IC50 value of 17.7 ± 5.1 µg/ml. Carboplatin had an IC50 value of 31.6 ± 8.1 µg/ml. This concentration of Galangin (IC50 17.7 ± 5.1) was utilized for all subsequent tests.

#### Apoptosis Regulation and the Role of Galangin

**Fig. 10:**
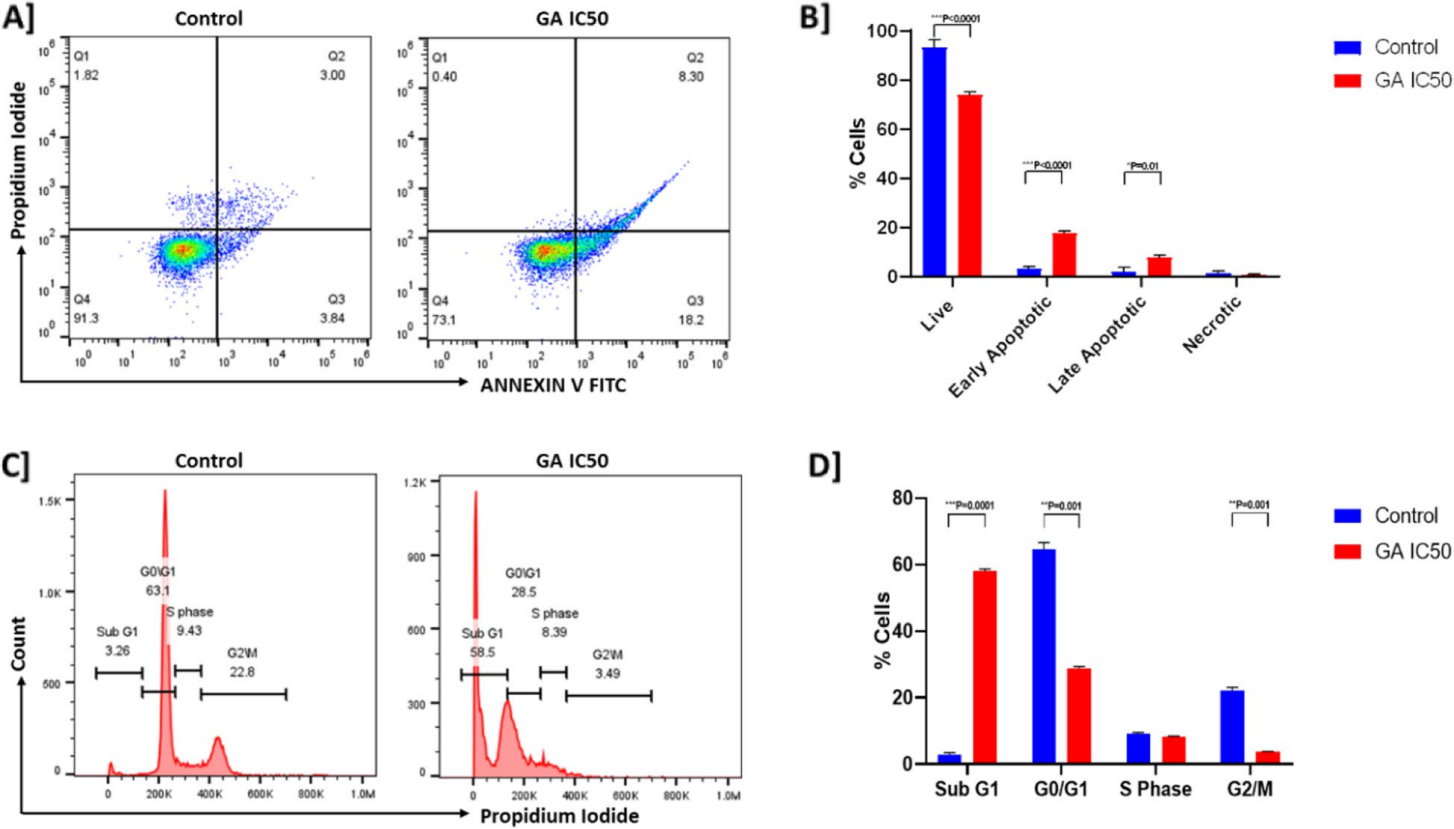
Effect of Galangin treatment on apoptosis and cell cycle in MDA-MB231cells. **A** Dot plot shows apoptosis ratios of MDA-MB231 cells in control (left) and after treatment of Galangin (right) for 24 h using propidium iodide and Annexin-V FITC. **B **Bar graph shows the percentages of live, early apoptotic, late apoptotic, and necrotic cells in control (blue) and after treatment ofGalangin (red) for 24 h. **C** Histogram represents different stages of the cell cycle in MDA MB231 cells in control (left) and after treatment of Galangin (right) for 24 h using propidium iodide. **D **Bar graph shows the percentages of cells in sub-G1, G0/G1, S, and G2/M phases in control (blue) and after treatment of Galangin (red) for 24 h. Error bar shows mean with standard deviation. *p < 0.05,**p < 0.01, ***p < 0.001

A 24-hour apoptosis investigation was conducted on MDA-MB231 cells using Galangin at its IC50 concentration. Figure 10A shows dot plots that illustrate how Galangin induces cell death. Figure 10B shows that compared to control cells, there is a significant decrease in viable cells (73.95 ± 1.2%) and an increase in early apoptotic cells (17.75 ± 0.6%) and late apoptotic cells (7.72 ± 0.8%). Galangin mostly causes cell death by inducing early apoptosis, according to these studies.

#### The impact of Galangin on the regulation of the cell cycle in MDA-MB231 cells

We performed an additional examination into the effects of Galangin therapy on the cell cycle of MDA-MB231 cells. Figure 10C displays sample graphs depicting different phases of the cell cycle in control cells compared to those treated with Galangin at dosages corresponding to its IC50 for 24 hours. Treatment with Galangin resulted in a significant increase in the proportion of cells in the Sub G1 phase (58.05 ± 0.6%), accompanied by a marked decrease in cells in the G0/G1 phase (28.85 ± 0.4%) and the G2/M phase (3.63 ± 0.2%), while the S-phase cell count remained stable (8.36 ± 0.03%), compared to control cells (Sub G1: 2.8 ± 0.5%, G0/G1: 64.55 ± 2.0%, S-phase: 9.25 ± 0.2%, G2/M: 22 ± 1.1%) (Fig. 10D). This study highlights the specific impact of Galangin in inhibiting cell cycle progression during the Sub G1 phase, suggesting its potential significance for therapeutic strategies aimed at apoptosis.

#### Impact of Galangin on intracellular reactive oxygen species (ROS) levels and mitochondrial membrane potential (ΔΨm) in MDA MB231 cells

**Fig. 11:**
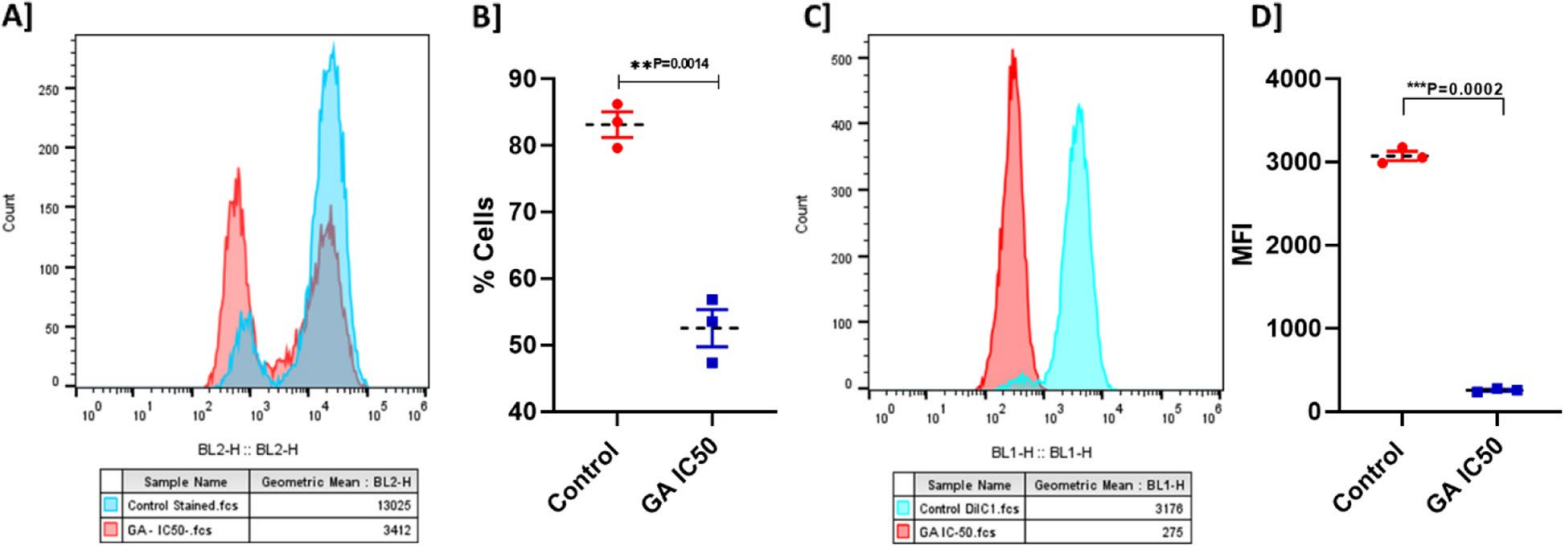
Impact of Galangin administration on reactive oxygen species production and mitochondrial membrane potential in MDA-MB231 cells. **A** The histogram illustrates the levels of reactive oxygen species (ROS) production prior to (blue) and following the administration of Galangin (red) in MDA MB231 cells. **B** The scatter dot plot illustrates the quantitative assessment of ROS generation prior to (red) and subsequent to (blue) the treatment with Galangin in MDA MB231 cells. **C** The histogram illustrates the mitochondrial membrane potential (ΔΨm) in MDA MB231 cells before (blue) and after treatment with Galangin (red). **D** The scatter dot plot illustrates the quantitative assessment of mitochondrial membrane potential in MDA MB231 cells prior to (red) and following (blue) Galangin therapy. The error bar represents the mean accompanied by the standard deviation. *p < 0.05, **p < 0.01, ***p < 0.001

Subsequently, we assessed the effect of Galangin on intracellular reactive oxygen species levels and mitochondrial membrane potential in MDA-MB231 cells. Figure 11A presents sample graphs demonstrating intracellular ROS levels prior to and following Galangin therapy, whereas Fig. 11C displays corresponding graphs for mitochondrial membrane potential. Post-Galangin therapy, a notable reduction in ROS levels (52.53 ± 4.82%) was recorded in contrast to the control (83.10 ± 3.31%) (Fig. 11B). Furthermore, Galangin administration resulted in a significant decrease in mitochondrial membrane potential (MFI: 256 ± 20.66) relative to the control (MFI: 3071 ± 96.46) (Fig. 11D).

### Activity of Galangin on Wound Scratch Assay

**Fig. 12:**
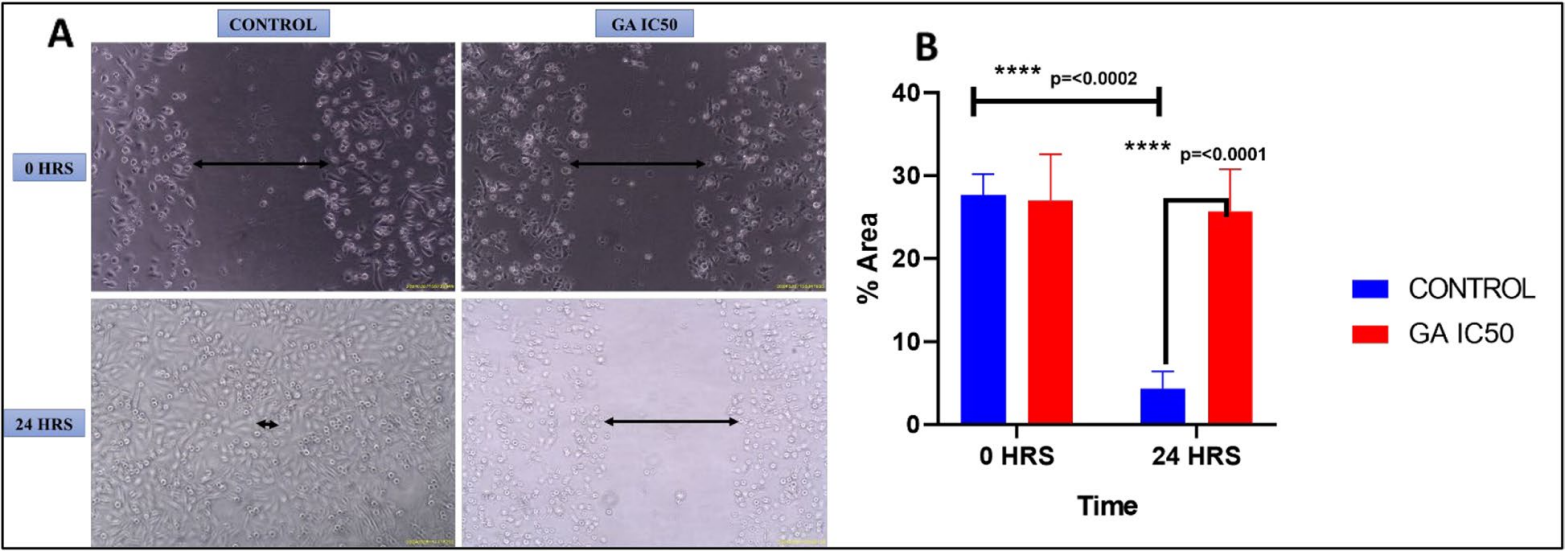
Illustrative Image for scratch assay. **A** MDAMB231 cells were treated with Galangin at IC50 (17.21 µg/ml) after 24 h. The cellular scratch was evaluated at 0 and 24 h utilizing a Magnus INVI microscope at × 100 magnification. The migration of MDAMB231 cells was inhibited by Galangin IC50 after 24 h, as illustrated in the second vertical panel. The initial panel depicted the control group. **B** The data were presented as mean ± standard deviation (SD), and “two-way ANOVA” was employed for analysis, followed by “Tukey” post hoc tests at ****p < 0.0001; ***p < 0.001; **p < 0.01 in comparison to the control at the corresponding time point

Following cell seeding, a 24-h incubation period was allowed prior to administering IC50 doses of Galangin. A control group was also established. The wells were evaluated at 0 and 24 h to determine the impact of the treatments. The results demonstrated that the control group displayed cell proliferation and migration after 24 h, which was absent in the Galangin-treated group (Fig. 12A). The Galangin-treated group exhibited a substantial reduction of cell proliferation and migration at 24 h in comparison to the control group (Fig. 12B).

### In MDA MB231 cells, galangin causes apoptosis by upregulating the expression of the BAX gene and downregulating that of the BAD gene

**Fig. 13:**
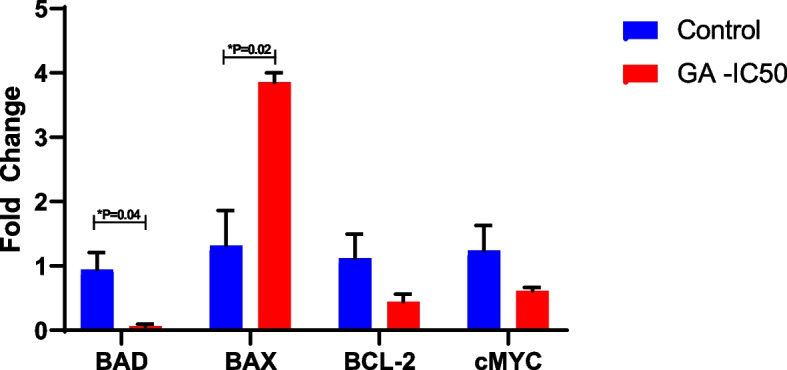
Effect of Galangin administration on the expression of several apoptosis-related genes. The bar graph illustrates the fold change in gene expression levels of BAD, BAX, BCL-2, and cMYC, prior to (blue) and subsequent to (red) the administration of Galangin in MDA-MB231 cells. The error bar represents the mean accompanied by the standard deviation. *p < 0.05, **p < 0.01, ***p < 0.001

Having identified Galangin-induced apoptosis in MDA MB231 cells, we continued to examine its effects on the expression levels of critical apoptosis-related genes: BAD, BAX, BCL-2, and cMYC. Our findings indicated a substantial reduction in BAD gene expression coupled with a marked elevation in BAX gene expression subsequent to Galangin administration. Moreover, Galangin decreased the expression levels of BCL-2 and cMYC relative to the control cells (Fig. 13). These findings indicate that Galangin may enhance apoptosis primarily by upregulating the BAX gene while downregulating the BCL-2 gene, thereby enhancing mitochondrial permeabilization. The IC50 value demonstrated a significant decrease in c-cMYC expression. This discovery indicates that Galangin may impede c-cMYC expression by attenuating TGF-β, therefore modulating cellular proliferation and death (Fig. 13). The IC50 value of Galangin significantly influenced the aforementioned gene expression, contributing to its anti-cancer efficacy against TNBC primarily as an apoptotic promoter and regulator of cellular proliferation.

## Discussion

Breast cancer (BC) manifests in diverse forms among women and can be categorized into three primary types based on molecular and histological characteristics: hormone receptor-positive BC (expressing estrogen receptor (ER +) or progesterone receptor (PR +)), human epidermal growth factor receptor 2-positive BC (HER2 +), and triple-negative breast cancer (TNBC) (ER − , PR − , HER2 −) [41]. Triple-negative breast cancer (TNBC) is the most aggressive and malignant subtype, characterized by a poor prognosis. Treatment modalities for TNBC are predominantly restricted to surgical intervention, chemotherapy, and radiation. Nevertheless, numerous patients receive a diagnosis too late for surgical intervention, complicating therapy endeavors [42].

Contemporary research increasingly emphasizes the investigation of alternative therapies with reduced toxicity, motivated by the unsatisfactory clinical results and considerable toxicity associated with current breast cancer treatments. The objective is to improve clinical outcomes while reducing drug adverse effects. There is an increasing interest in exploring complementary and alternative medicine as a viable option for cancer management. The current research endeavor concentrates on investigating novel natural compounds for cancer treatment, utilizing both in silico analysis and in vitro methodologies to forecast and confirm their efficacy against certain tumors [43–45].

In this study, we first performed in silico analysis and then in vitro experiments were performed. This study’s in silico research identified 40 common genes linked to Galangin and triple-negative breast cancer, involved in critical biological processes like the PI3K-Akt signaling cascade, cancer pathways, proteoglycans in cancer, and microRNAs in cancer. During the PPI study, it was discovered that there was a considerable impact on cellular proliferation and apoptosis, in addition to an influence on the cell cycle that had an effect on the G1 and G2M transitions. Our investigations indicated that Galangin exhibited an anti-apoptotic activity with a sub-G1 phase arrest of the cell cycle phenomena. The findings of the in silico study were supported by the in vitro studies, and the in vitro studies validated the findings of the in silico study. EGFR, PIK31, MET, GSK3B, SRC, and CDK6 were identified as prominent hub genes, consistently associated with the top 10 pathways. The PI3K-Akt signaling pathway is a crucial mechanism for the possible therapeutic effects of Galangin on breast cancer. In breast cancer tissues, increased mRNA levels of ESR1, PARP1, GSK3B, and MMP9 were detected. The molecular docking studies indicated that the binding energies and schematic representations of PTGS2 (− 9.8) and SRC (− 9.5) demonstrated a robust interaction and efficacy of Galangin. This indicates that Galangin exerts its effects via the PI3K-Akt pathway, which should be validated by the in vitro studies. No in silico studies on Galangin’s efficacy in treating triple-negative breast cancer have been conducted prior to this research. Nonetheless, numerous in vitro research exists.

In the present investigation, Galangin demonstrated substantial cytotoxicity with an IC50 value of 17.7 µg/ml, inducing cell death via early apoptosis at this dose. These findings align with prior studies that emphasized the apoptotic and antiproliferative properties of Galangin in relation to breast cancer, specifically triple-negative breast cancer (TNBC). Moreover, an elevation in the formation of reactive oxygen species (ROS) was noted in these investigations, hence reinforcing its mechanism of action. Xuan et al. found seven active constituents in Chinese Propolis water extract, including Galangin, which had antiproliferative effects on various tumor cell lines by enhancing ROS production and activating caspase 3 to induce apoptosis [46]. The current study demonstrated that Galangin reduces ROS, indicating antioxidant action. Vukovic et al. demonstrated that Galangin and other flavonoids derived from Propolis had notable antiproliferative, proapoptotic, and antioxidative effects on colon and breast cancer cell lines, with Galangin specifically triggering considerable apoptosis in MDA-MB-231 cells. Galangin exhibited significant pro-apoptotic activity in the MDA-MB-231 cell line, inducing 34.29% of early and late apoptosis at a dose of 10 μM [47]. Noureddine et al. conducted a study employing in vitro techniques to ascertain the cytotoxic and antiproliferative properties of Lebanese propolis, predominantly consisting of Galangin. It has shown significant efficacy against many cancer cells, including Jurkat leukemia T-cells, glioblastoma U251 cells, and breast adenocarcinoma MDA-MB-231 cells [48]. The anti-proliferative action aligned with our study findings. Furthermore, Arif et al. emphasized the pro-oxidant mechanism of flavonoids, such as Galangin, in promoting apoptosis and cytotoxicity in cancer cells through copper-dependent DNA fragmentation and the generation of reactive oxygen species, thus offering a targeted chemopreventive approach against cancer [49]. Liu et al. discovered that in MCF-7 cells, Galangin enhances the expression of the pro-apoptotic protein Bax while diminishing the expression of the anti-apoptotic protein Bcl-2 in a concentration-dependent manner. These findings aligned with our results.

Murray et al. similarly found that Galangin strongly suppresses the transition from the G0/G1 phase to the S phase, principally via downregulating cyclin D3 and suppressing cyclins A and E. This result leads to significant inhibition of AhR activity and cellular proliferation in Hs578T breast cancer cells. This distinctive mechanism highlights Galangin’s potential as a chemotherapeutic agent, especially when combined with treatments that address other facets of the tumor cell cycle, and in instances when estrogen receptor-specific therapies prove ineffective [50]. The current study demonstrated an increase of cells in the sub-G1 phase, indicating apoptosis.

Notwithstanding the robust in vitro evidence supporting the chemopreventive and chemotherapeutic attributes of this drug, further in vivo investigations utilizing various tumor models are unequivocally required in the future. The safety concerns of Galangin must be addressed before proceeding with the clinical trials. Finally, it is anticipated that contemporary nanotechnological techniques will identify the optimal carrier systems for effectively delivering sufficient quantities of the parent bioactive compound Galangin to the targeted cancerous tissues.

## Future perspective

The in silico study identified hub genes and important pathways targeted by Galangin in triple-negative breast cancer, primarily the PI3K-AKT and MAPK pathways. Previous research has shown the antiproliferative, apoptotic, and antimigratory properties of Galangin; our investigations sought to corroborate these findings, particularly regarding apoptosis, in accordance with our in silico predictions. Additional study is required to clarify the processes of Galangin via the anticipated pathways and genes. This necessitates the validation of these findings through the execution of many in vitro and in vivo research.

## Conclusion

The research illustrates the potential effectiveness of Galangin against triple-negative breast cancer, influencing various targets and pathways. Molecular docking methods demonstrate advantageous interactions with critical targets. The utilization of in silico analysis has improved our comprehension of Galangin’s mechanism of action, offering a thorough evaluation across multiple criteria specified in this methodology. Galangin demonstrates potential as an effective drug against TNBC by regulating cell growth and death. Further research is required to validate the clinical efficacy of Galangin and to clarify its underlying processes in relation to TNBC.

## Supplementary Information

Supplementary Material 1.

## Data Availability

No datasets were generated or analysed during the current study.

## Abbreviations

BC: Breast Cancer
Carbo: Carboplatin
CDK: Cyclin Dependent Kinase
DAVID: Database for Annotation, Visualization, and Integrated Discovery
DisGeNet: Disease Gene Network
GA: Galangin
GEPIA: Gene Expression Profiling Interactive Analysis
GO: Gene Ontology
KEGG: Kyoto Encyclopaedia of Genes and Genome
MCC: Maximal Clique Centrality
MDA-MB-231: M.D. Anderson Metastasis Breast cancer (TNBC cell line)
ΔΨm: Mitochondrial Membrane Potential
MTT: 3-[4,5-dimethylthiazol-2-yl]-2,5 diphenyl tetrazolium bromide
PPI: Protein-Protein Interaction
ROS: Reactive Oxygen Species
STRING: Search Tool for the Retrieval of Interacting Genes/Proteins
SwissADME: Swiss Adsorption, Distribution, Metabolism, Excretion
TNBC: Triple-negative Breast Cancer
