# Supplementary material for: Galangin promotes apoptosis by upregulating the pro-apoptotic gene BAX in triple-negative breast cancer
Source: J Egypt Natl Canc Inst. 2024 Dec 20;36:41. doi: 10.1186/s43046-024-00246-y (PMC13313835; doi:10.1186/s43046-024-00246-y)
Supplement: Supplementary file 1 — Supplementary Material 1. [file 43046_2024_246_MOESM1_ESM.docx]

**Supplementary Table [ST]**

**ST:**  Primer Sequences of genes used in the study.

| Gene NAME | SEQUENCE (5'->3') |
| --- | --- |
| BAX - F | GGTTGTCGCCCTTTTCTA |
| BAX - R | CGGAGGAAGTCCAATGTC |
| BAD - F | GGAGCATCGTTCAGCAGCAG |
| BAD - R | CCATCCCTTCATCTTCCTCAGTC |
| BCL 2 - F | TTGCTTTACGTGGCCTGTTTC |
| BCL 2 – R | GAAGACCCTGAAGGACAGCCAT |
| Beta Actin- F | AGAGCTACGAGCTGCCTGAC |
| Beta Actin- R | AGCATTTCTTCCCGGCCTTT |
| c-MYC- F | AGAAATGTCCTGAGCAATCACC |
| c-MYC- R | AAGGTTGTGAGGTTGCATTTGA |
